# Subinhibitory Concentrations of Thymol Reduce Enterotoxins A and B and α-Hemolysin Production in *Staphylococcus aureus* Isolates

**DOI:** 10.1371/journal.pone.0009736

**Published:** 2010-03-17

**Authors:** Jiazhang Qiu, Dacheng Wang, Hua Xiang, Haihua Feng, Youshuai Jiang, Lijie Xia, Jing Dong, Jing Lu, Lu Yu, Xuming Deng

**Affiliations:** 1 Institute of Zoonoses, College of Animal Sciences and Veterinary Medicine, Jilin University, Changchun Jilin, People's Republic of China; 2 College of Life Science and Technology, Heilongjiang August First Agricultural University, Daqing Heilongjiang, People's Republic of China; 3 College of Veterinary Medicine, Yangzhou University, Yangzhou Jiangsu, People's Republic of China; National Institutes of Health, United States of America

## Abstract

**Background:**

Targeting bacterial virulence factors is now gaining interest as an alternative strategy to develop new types of anti-infective agents. It has been shown that thymol, when used at low concentrations, can inhibit the TSST-1 secretion in *Staphylococcus aureus*. However, there are no data on the effect of thymol on the production of other exotoxins (e.g., α-hemolysin and enterotoxins) by *S. aureus*.

**Methodology/Principal Findings:**

Secretion of α-hemolysin, SEA and SEB in both methicillin-sensitive and methicillin-resistant *S. aureus* isolates cultured with graded subinhibitory concentrations of thymol was detected by immunoblot analysis. Hemolysin and tumor necrosis factor (TNF) release assays were performed to elucidate the biological relevance of changes in α-hemolysin, SEA and SEB secretion induced by thymol. In addition, the influence of thymol on the transcription of *hla*, *sea*, and *seb* (the genes encoding α-hemolysin, SEA and SEB, respectively) was analyzed by quantitative RT-PCR. Thymol inhibited transcription of *hla*, *sea* and *seb* in *S. aureus*, resulting in a reduction of α-hemolysin, SEA and SEB secretion and, thus, a reduction in hemolytic and TNF-inducing activities.

**Conclusions/Significance:**

Subinhibitory concentrations of thymol decreased the production of α-hemolysin, SEA and SEB in both MSSA and MRSA in a dose-dependent manner. These data suggest that thymol may be useful for the treatment of *S. aureus* infections when used in combination with β-lactams and glycopeptide antibiotics, which induce expression of α-hemolysin and enterotoxins at subinhibitory concentrations. Furthermore, the structure of thymol may potentially be used as a basic structure for development of drugs aimed against these bacterial virulence factors.

## Introduction

*Staphylococcus aureus* is a leading cause of both community- and hospital-acquired infections associated with significant morbidity and mortality. This pathogen causes a wide spectrum of clinical illnesses, including skin and soft tissue lesions, and lethal infections such as osteomyelitis, endocarditis, pneumonia and septicemia [1]. The continuous emergence of methicillin-resistant *S. aureus*, glycopeptide-insensitive *S. aureus* (GISA) and vancomycin-resistant *S. aureus* strains (VRSA) has made it difficult to treat *S. aureus* infections [2]. Moreover, it is well known that *S. aureus* can secrete a number of exotoxins (e.g., hemolysins, enterotoxins, coagulase, TSST-1 and protein A), which contributes to the ability of this pathogen to cause such a variety of diseases [3].

α-hemolysin and enterotoxins are major virulence factors secreted by *S. aureus* strains. α-hemolysin is a 33-kDa pore-forming protein that has cytolytic, hemolytic and dermonecrotic activities. A wide range of human cells, including erythrocytes, monocytes, lymphocytes, macrophages and epithelial cells, are affected by α-hemolysin. Staphylococcal enterotoxins (SEs) are the virulence factors responsible for staphylococcal gastroenteritis and are one cause of food poisoning in humans. The enterotoxins also have the immunomodulatory properties of superantigens, stimulating release of T-cell-derived cytokines and T-cell activation [4]. To date, a number of SEs have been identified, including SEA-E, SEG, SEH, SEI, SEJ, SEK, SEL, SEM, SEN and SEO [5].

Like most staphylococcal exoproteins, α-hemolysin and SEs are not expressed constitutively, but are primarily secreted during the post-exponential growth phase [3]. Furthermore, the expression of virulence factors is generally modulated in response to alterations in cell-population density through a process referred to as quorum sensing [6].

Thymol, a *p*-cymene-derived compound primarily found in thyme, oregano and tangerine peel, is widely used in medicine for its multiple biological properties, such as its anti-oxidant, anti-bacterial, anti-leishmanial and hepatoprotective activities [7]–[10]. Furthermore, we previously reported that thymol has antifungal activity against clinical isolates of fluconazole-sensitive and -resistant *Candida albicans*, and investigated the effect of thymol on global gene expression of the model fungus *Saccharomyces cerevisiae* [11], [12]. It has been demonstrated that thymol is active against *S. aureus* and can suppress the TSST-1 secretion in *S. aureus* when used at low concentrations that minimally affect bacterial growth [13], [14]. However, to our knowledge, the effects of thymol on secretion of α-hemolysin and enterotoxins by *S. aureus* remain uncharacterized.

The goal of this study was to investigate the effect of subinhibitory concentrations of thymol on the expression of α-hemolysin and two major enterotoxins (SEA and SEB) by methicillin-sensitive *S. aureus* (MSSA) and methicillin-resistant *S. aureus* (MRSA).

## Results

### Growth of *S. aureus* with subinhibitory concentrations of thymol

**Figure 1:**
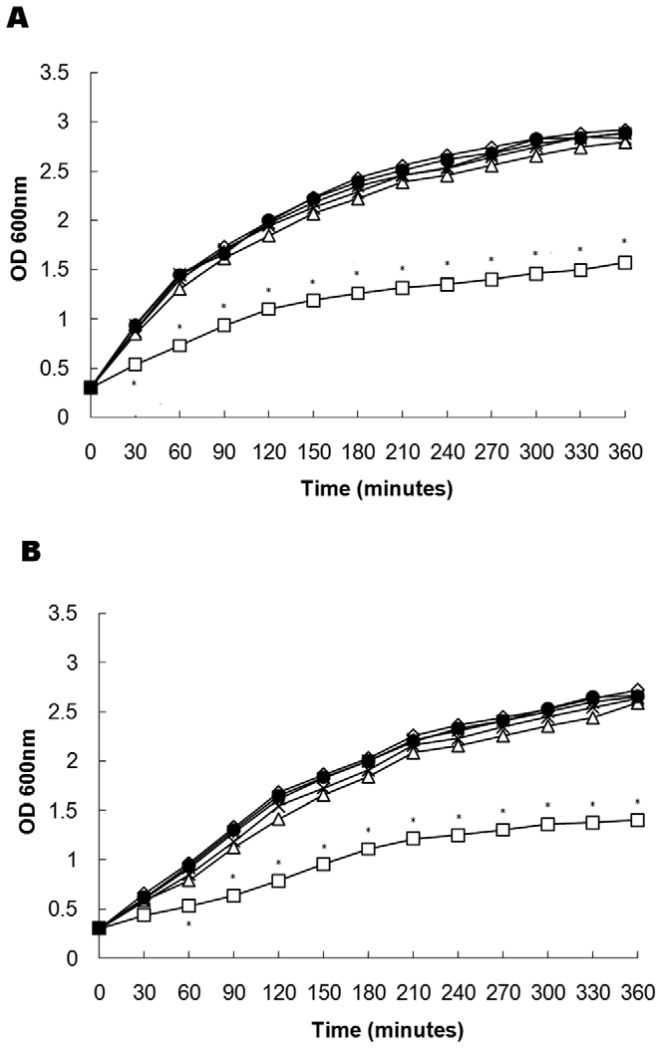
Growth curve for *S. aureus* ATCC 29213 (A) and MRSA strain 2985 (B). (◊), untreated *S. aureus*; (•), *S. aureus* cultured with 8 µg/ml thymol; (*), *S. aureus* cultured with 16 µg/ml thymol; (×), *S. aureus* cultured with 32 µg/ml thymol; (△), *S. aureus* cultured with 64 µg/ml thymol; (□), *S. aureus* cultured with 128 µg/ml thymol. Values are the averages of three independent experiments. * represents *p*<0.05.

The minimum inhibitory concentrations (MICs) of thymol against 29 *S. aureus* strains were determined and ranged from 64 to 256 µg/ml. The MIC values of thymol against *S. aureus* ATCC 29213 and MRSA strain 2985 were 128 µg/ml. These results indicate that the thymol structure could be an important basic structure for development of novel anti-*S. aureus* drugs. The growth curves of *S. aureus* ATCC 29213 cultured with graded subinhibitory concentrations of thymol are shown in Fig. 1A; Thymol, at levels from 1/16×MIC to 1/2×MIC had no significant effects on the growth of *S. aureus*. However, during culture with 1×MIC of thymol, the growth rate was significantly decreased; after 30, 180 and 360 min of thymol treatment, OD_600_ values were 57.2%, 51.8% and 53.6% of the control culture, respectively. The growth of MRSA strain 2985 was affected by these concentrations of thymol in similar manner (Fig. 1B).

### Influence of thymol on α-hemolysin, SEA and SEB production by *S. aureus*

**Figure 2:**
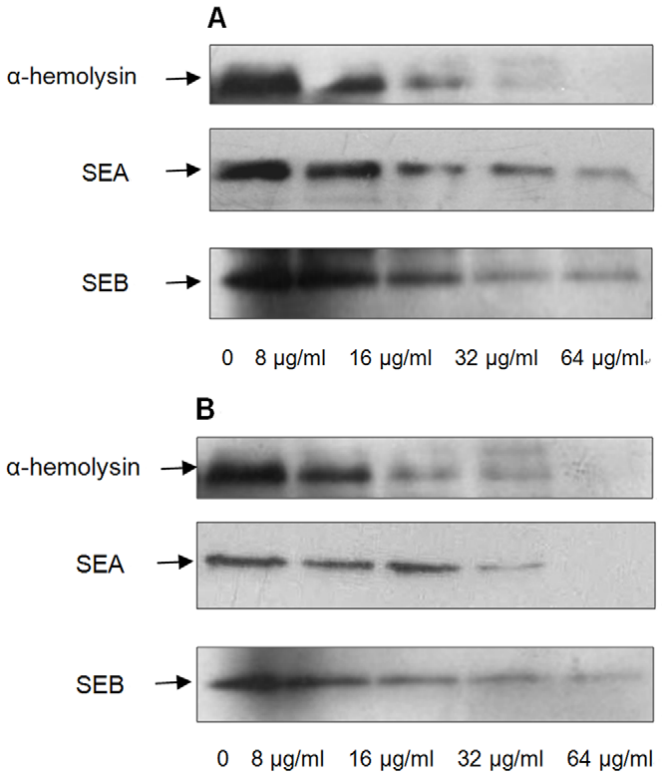
Western blot analysis of α-hemolysin, SEA and SEB production. Both strain ATCC 29213 (A) and MRSA 2985 (B) were cultured with graded subinhibitory concentrations of thymol to an OD_600_ of 2.5. Supernatants were subjected to SDS-PAGE. After transfer to polyvinylidene fluoride membranes, proteins were stained with the indicated antibodies against α-hemolysin, SEA and SEB. Horseradish peroxidase-conjugated goat anti-rabbit antiserum was used as secondary antibody, and the blots were developed using the ECL substrate (GE Healthcare, UK).

Western blot assays were performed to detect the levels of α-hemolysin, SEA and SEB produced by *S. aureus*. As exoproteins are principally secreted during post-exponential growth, *S. aureus* ATCC 29213 and MRSA 2985 were cultured with graded subinhibitory concentrations of thymol to an OD_600_ of 2.5. As shown in Fig. 2, treatment with thymol resulted in a dose-dependent decrease in the secretion of α-hemolysin, SEA and SEB. Culture with 1/16 × MIC of thymol resulted in a recognizable reduction in secretion of α-hemolysin, SEA and SEB; during culture with 1/2 × MIC, little or no immunoreactive proteins could be detected in strains ATCC 29213 and MRSA 2985.

The apparent reduction in secretion of α-hemolysin, SEA and SEB could result from an increase in protease secretion by *S. aureus* cultured in thymol-containing medium; to address this possibility, extracellular proteases were quantified using azocasein. There was no significant effect on protease secretion by ATCC 29213 or MRSA 2985 cultured with 1/2 × MIC of thymol (data not shown).

### Thymol attenuates hemolytic and TNF-inducing activities of *S. aureus* supernatants

It has been shown that secretion of α-hemolysin by *S. aureus* results in hemolysis of rabbit erythrocytes, while enterotoxins act as superantigens, stimulating T-cells to release proinflammatory cytokines (e.g., TNF-α). Therefore, hemolysin and tumor necrosis factor (TNF) release assays were performed to elucidate the biological relevance of the reduction in α-hemolysin, SEA and SEB secretion induced by thymol.

When cultured with 1/16 × MIC of thymol, hemolysis of ATCC 29213 and MRSA 2985 culture supernatants were 62.2% and 70.5% compared to respective control culture, respectively. Notably, during culture with 1/4 × MIC or 1/2 × MIC of thymol, no hemolysis was observed (Table 1); this dose-dependent attenuation of hemolysis was observed in both strains. However, treatment with thymol did not directly cause hemolysis of rabbit erythrocytes at 1 × MIC or 2 × MIC concentrations. Additionally, there was little influence on the hemolytic activity of culture supernatants when pre-incubated with a 2 × MIC of thymol (data not shown). Although α-hemolysin is a major hemolysin, *S. aureus* contains other hemolytic toxins, such as β- and γ-hemolysin. Therefore, not all hemolytic activity is due to α-hemolysin. From the results of hemolysin assays, it is reasonable to infer that the production of other hemolytic toxins in *S. aureus* may also be inhibited by subinhibitory concentrations of thymol.

**Table 1:** Hemolytic activities of α-hemolysin produced by *S. aureus* cultured with graded subinhibitory concentrations of thymol.

Strains	Hemolysis (%) of rabbit erythrocytes by culture supernatant^a^				
	0	8 µg/ml	16 µg/ml	32 µg/ml	64 µg/ml
ATCC 29213	100%	62.2%±4.9	14.1%±3.5**	NO.	NO.
MRSA 2985	100%	70.5%±6.2	26.5%±4.2*	NO.	NO.

*^a^*The drug-free culture supernatants served as 100% hemolysis.

NO. means no hemolytic activity was observed.

Values represent the mean and standard error of three independent experiments.

* , *p*<0.05 and

** , *p*<0.01 compared to the corresponding control.

**Figure 3:**
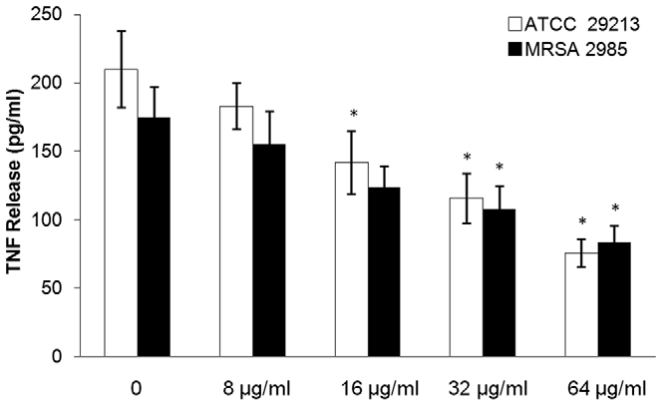
TNF release from splenocytes stimulated with supernatants of *S. aureus*. Spleen cells were stimulated with supernatants of *S. aureus* previously grown to an OD_600_ of 2.5 in the absence or presence of graded subinhibitory concentrations of thymol in RPMI-1640. After 16 h of stimulation, TNF release was measured by ELISA. Values represent the mean ± SD for three independent experiments. * indicates statistically significant reduction in TNF release (*p*<0.05).

Culture supernatants of *S. aureus* treated with graded subinhibitory concentrations of thymol elicited reduced amounts of TNF-α produced by spleen cells (Fig. 3). However, thymol did not directly reduce TNF release at 1×MIC or 2×MIC concentrations. Thymol diminished the TNF-inducing activity of *S. aureus* supernatants in a dose-dependent manner.

### Thymol diminishes the transcription of *hla*, *sea*, *seb* and *agrA* in *S. aureus*

**Figure 4:**
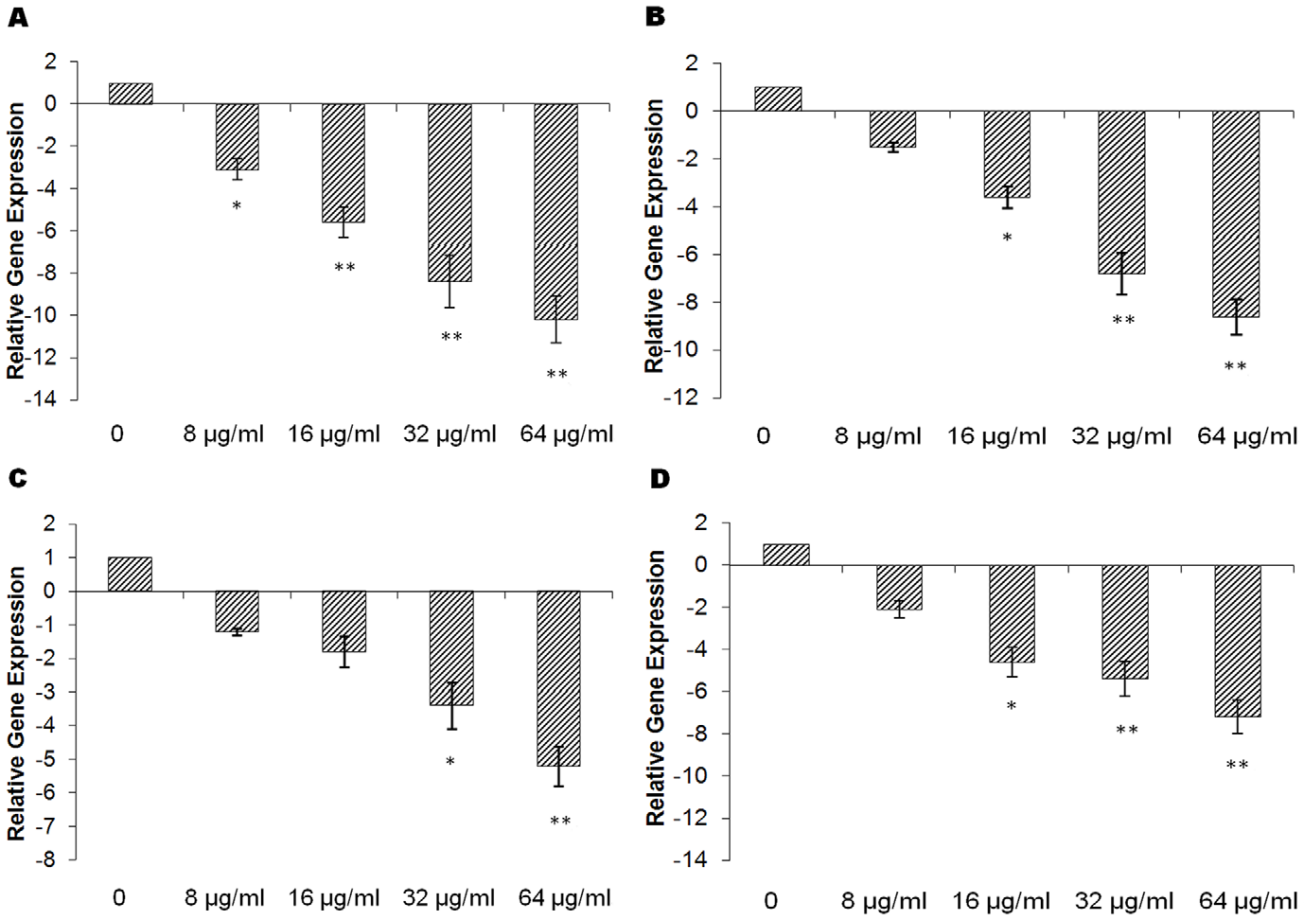
Relative expression of *hla* (A), *sea* (B), *seb* (C) and *agrA* (D) in *S. aureus*. *S. aureus* ATCC 29213 was cultured with different subinhibitory concentrations of thymol to the post-exponential growth phase (t = 240 min). Transcript levels were monitored by quantitative RT-PCR as described in the Materials and Methods. The drug-free culture was used as calibrator. Values represent the mean ± SD for three independent experiments. * represents *p*<0.05 and ** represents *p*<0.01.

Since thymol significantly decreased the production of α-hemolysin, SEA and SEB by *S. aureus*, we hypothesized that thymol could affect transcription of *hla*, *sea* and *seb*. Quantitative RT-PCR was performed to assess relative expression levels of *hla*, *sea* and *seb*. Since the these genes are positively regulated by the Agr two-component system [15], the transcription of *agrA* was also investigated. A dose-dependent reduction of *hla*, *sea*, *seb* and *agrA* transcription was observed in the *S. aureus* strain ATCC 29213 upon treatment with thymol (Fig. 4). For example, when cultured with 1/2 × MIC concentration of thymol, the transcriptional levels of *hla*, *sea*, *seb* and *agrA* were decreased by 10.2-, 8.6-, 5.2 and 7.2-fold, respectively.

## Discussion

The dramatic increase in methicillin-resistant *S. aureus*, in addition to the emergence of vancomycin-resistant isolates, has accelerated and broadened the interest in the development of new therapeutics to aid in the prevention and treatment of life-threatening infections caused by these strains [16]. Currently, there is a focus on identifying natural compounds that may serve as basic structures for the development of novel anti-*S. aureus* drugs [17]. Thymol, the major phenolic component of oregano oil, is known for its wide spectrum of antimicrobial activity and has been the subject of several investigations [18]. In this study, we have demonstrated that thymol is active against both MSSA and MRSA; therefore, thymol may potentially be used as a lead compound for the design of more potent antibacterial agents that could be used to fight drug-resistant *S. aureus* strains.

An alternate strategy that is gaining great interest is treatment that targets bacterial virulence factors (e.g., enterotoxins, hemolysins and adhesins) [19], [20]. The pathogenicity of *S. aureus*, similar to that of other Gram-positive bacteria, to a great extent, depends on the production of numerous extracellular virulence factors. Consequently, the clinical performance of antibiotics is not only determined by their bactericidal or bacteriostatic activity, but also by their influence on virulence factor release [21]. Many antibiotics affect the secretion of exotoxins by *S. aureus*, especially at suboptimal concentrations. Inhibitors of protein synthesis, such as linezolid and clindamycin, significantly inhibit the production of virulence factors (including α-hemolysin, SEA, SEB and protein A) in *S. aureus* at subinhibitory concentrations [22], [23]. In contrast, bactericidal antibiotics that affect the cell wall (for example, β-lactams and glycopeptides), induce the expression of α-hemolysin, enterotoxins and TSST-1 through a stimulatory effect on exoprotein synthesis [24]. It has also been demonstrated that some plant compounds (e.g., oleuropein and epicatechin gallate) and essential oils (e.g., oils of bay, cinnamon and clove) can influence production of exotoxins when used at subinhibitory concentrations [17], [25], [26]. The antibiotic-induced regulation of virulence factors may result in either aggravation or attenuation of the infection. Therefore, the regulation of toxin secretion is extremely significant for diseases caused by *S. aureus*, and the ability of antibiotics to affect these properties may be an important criterion in selecting an antibiotic for therapy [27].

Many studies have indicated that, in addition to drug resistance, *S. aureus* has the ability for increased virulence, which may lead to more severe and widespread disease. For instance, Li and colleagues reported that community-associated MRSA USA300 and its progenitor USA500 were clearly distinct from other MRSA strains. These strains displayed high virulence in animal infection models and had the capacity to evade innate host defense mechanisms. The increased virulence in the USA300/USA500 sublineage was attributed to differential expression of core genome-encoded virulence determinants, such as phenol-soluble modulins and α-toxin [28]. Holden *et al*. have also provided evidence for the rapid evolution of virulence by comparing complete genomes of two clinical *S. aureus* strains [29]. Furthermore, it has been demonstrated that virulence factors can transfer between *S. aureus* and *Staphylococcus epidermidis*, which may result in the transformation of *S. epidermidis* from a commensal organism into a more aggressive opportunistic pathogen [30]. Taken together, these findings highlight the significance and urgency of developing antimicrobial agents which target virulence factors.

In the present study, we have shown by transcriptional, expressional and phenotypic analyses that subinhibitory concentrations of thymol reduce α-hemolysin, SEA and SEB secretion in both MSSA and MRSA in a dose-dependent manner. These results indicate that thymol may be useful for the treatment of *S. aureus* infections when used in combination with β-lactams and glycopeptide antibiotics, which could induce expression of α-hemolysin and enterotoxins at subinhibitory concentrations. Additionally, the structure of thymol may be used as a basic structure for development of drugs aimed at the bacteria virulence factors.

In view of the increasing incidence of antibiotics resistance, there is also a continuing need to find more and improved antimicrobial agents in the food industry. The SEs preformed within the food could cause serious food-borne diseases, including staphylococcal gastroenteritis and food poisoning. Furthermore, SEs are a family of major serological types of heat stable enterotoxins [31]. Consequently, the ability of thymol to decrease the production of SEA and SEB adds impetus to its potential as a novel food preservative.

The expression of most virulence factors by *S. aureus* is regulated by a network of interacting regulators, such as *agr*, *sar* and *sae* [32]. Previous reports indicated that subinhibitory concentrations of antibiotics may interfere with translation of one or more regulatory gene products in *S. aureus*, and therefore may affect transcription of exoprotein-encoding genes. For example, subinhibitory concentrations of clindamycin differentially inhibited the transcription of exoprotein genes in *S. aureus* and acted partly through *sar* [22]; subinhibitory concentrations of β-lactams induced hemolytic activity in *S. aureus* through the SaeRS two-component system [33]. In this study, quantitative RT-PCR was used to investigate the influence of thymol on the *agr* locus of *S. aureus*. Our results showed that thymol significantly inhibited *agrA* transcription. However, the mechanisms by which *S. aureus* controls virulence gene expression are fairly intricate and involve an interactive, hierarchical regulatory cascade among the products of the *sar*, *agr*, and other components [34]. Therefore, we presume that the reduction of virulence factor production observed in our study may partially depend on thymol-induced inhibition of the Agr two-component system.

In conclusion, considering the broad-spectrum antimicrobial activities of thymol, as well as the findings reported in this study, we propose that thymol could potentially be used in the pharmaceutical and food industries.

## Materials and Methods

### Ethics statement

All animal studies were conducted according to the experimental practices and standards approved by the Animal Welfare and Research Ethics Committee at Jilin University. The study was also approved by the Institutional Review Board of the First Hospital of Jilin University, and all patients provided written informed consent for the collection of samples and subsequent analysis.

### Bacterial strains and reagents

MSSA strain ATCC 29213 was obtained from the American Type Culture Collection (ATCC). 28 clinical *S. aureus* isolates (15 MSSA and 13 MRSA) were isolated at the First Hospital of Jilin University from blood samples of infected patients; the clinical MRSA strain 2985, which has the ability to secrete α-hemolysin, SEA and SEB, was chosen for further experiments. Luria-Bertani (LB) broth was purchased from BD Biosciences, Inc. (Sparks, MD). Thymol was purchased from Sigma-Aldrich (St. Louis, MO), and dilutions were prepared in dimethyl sulfoxide (DMSO) (Sigma-Aldrich, St. Louis, MO).

### Determination of antibiotic MIC

MICs of thymol for *S. aureus* were evaluated in triplicate by a broth microdilution method as recommended by the Clinical and Laboratory Standards Institute [35]. The MIC was defined as the lowest concentration at which no visible growth was observed.

### Growth curves

*S. aureus* strain ATCC 29213 was cultured in LB to an OD_600_ of 0.3, and 100-ml volumes were aliquoted into six 500-ml Erlenmeyer flasks. Thymol (dissolved in DMSO) was added to five of the cultures to obtain final concentrations of 1/16 × MIC (8 µg/ml), 1/8 × MIC (16 µg/ml), 1/4 × MIC (32 µg/ml), 1/2 × MIC (64 µg/ml), or 1 × MIC (128 µg/ml). The final DMSO concentration for all conditions was 1% (v/v). A control culture received 1% DMSO only. Following addition of thymol (or DMSO), bacteria were cultured at 37°C with aeration and cell growth was monitored spectrophotometrically by measuring the OD_600_ at 30 min intervals. Three-ml samples of each culture were collected immediately after the addition of thymol (t_0_) and after 30, 60, 90, 120, 150, 180, 210, 240, 270, 300, 330 and 360 min.

### Western blot assay

Bacteria were cultured in LB containing subinhibitory concentrations of thymol to post-exponential growth phase (OD_600_ of 2.5, equivalent to 1×10^9^ CFUs/ml). Supernatants were collected by centrifugation, and the whole cells were removed through a 0.2-µm filter. A 25 µl volume of the supernatant was used for determination of α-hemolysin, SEA and SEB. Western blot analysis was performed under the conditions described by Towbin [36]. Antibodies to α-hemolysin, SEA and SEB were purchased from Sigma-Aldrich.

### Hemolysin assay

Bacteria were cultured in LB with graded subinhibitory concentrations of thymol to the post-exponential growth phase (OD_600_ of 2.5, equivalent to 1×10^9^ CFUs/ml). α-hemolysin levels in bacterial culture supernatants were determined based on the method of Rowe and Welch [37]. Briefly, bacterial samples (0.5 ml) were centrifuged (5,500×g, 4°C, 1 min), the supernatant was removed and 0.1 ml of supernatant was brought up to 1 ml in hemolysin buffer (0.145 M NaCl, 0.02 M CaCl_2_). After incubation with 25 µl of defibrinated rabbit blood (Biotech, Ltd., Changchun, China) for 30 min at 37°C, samples were centrifuged (5,500×g, room temperature, 1 min) and the optical densities of the supernatants were measured at 543 nm.

### Tumor necrosis factor (TNF) release assay

The TNF release assay was performed as described by Bernardo with some modification [23]. Overnight cultures of MSSA ATCC 29213 and MRSA 2985 in RPMI-1640 (Invitrogen, CA, USA) were diluted 30-fold in 500 ml of prewarmed RPMI-1640. Following incubation for 30 min at 37°C with aeration, cultures were divided into 100-ml aliquots. Graded concentrations of thymol (1/16, 1/8, 1/4 and 1/2 × MIC) were added to bacterial suspensions; following the addition of thymol, cultures were incubated for an additional 4 h. The final DMSO concentration for all conditions was 1% (v/v). The control culture was treated with 1% DMSO only. *S. aureus* supernatants without antibiotic treatment served as controls. Proteins secreted into the supernatants were filtered through a 0.2-µm filter and immediately analyzed as described below.

Specific-pathogen-free BALB/c mice (male, 6 to 8 weeks old, weighing 18 to 22 g) were obtained from the Experimental Animal Center of Jilin University (Changchun, China). Spleen cell suspensions were prepared in RPMI-1640, washed and resuspended in complete RPMI-1640 medium (RPMI-1640 media supplemented with 10% fetal bovine serum, 2 mM glutamine, 100 IU/ml penicillin, 100 IU/ml streptomycin, 15 mM HEPES and 50 µM 2-mercaptoethanol). A total of 10^6^ (150 µl) cells were dispensed into wells of a 96-well tissue culture plate. 50 µl of *S. aureus* culture supernatants were added to the tissue culture plate. After incubation for 16 h, supernatants were collected, centrifuged (1,000×g for 5 min) and TNF in the supernatants was determined by using the Mouse TNF-α ELISA MAX™ Set Standard (Biolegend, Inc., San Diego, USA), according to the instructions of the manufacturer.

### Quantitative RT-PCR

Strain ATCC 29213 was cultured in LB with graded subinhibitory concentrations of thymol to the post-exponential growth phase (t = 240 min). RNA was prepared as described by Sambanthamoorthy [38]. Briefly, cells were harvested by centrifugation (5,000×g for 5 min at 4°C) and resuspended in TES buffer containing 100 µg/ml lysostaphin (Sigma-Aldrich). The samples were incubated at 37°C for 10 min and then applied to a Qiagen RNeasy Maxi column to isolate total bacterial RNA, according to the manufacturer's directions. The optional on-column RNase-free DNase I (Qiagen, Hilden, Germany) treatment was performed to remove contaminating DNA. After isolation of RNA, traces of contaminating DNA were further eliminated by treating RNA samples with RNase-free DNase I (Ambion, Austin, TX) at 37°C for 20 min. Samples were either used immediately or stored at −80°C. The quality, integrity, and concentration of RNA were determined by using an Agilent 2100 Bioanalyzer (Agilent Technologies, Palo Alto, Calif.) as described by the manufacturer. The primer pairs used in quantitative RT-PCR are listed in Table 2. RNA was reverse transcribed into cDNA using the Takara RNA PCR kit (AMV) Ver. 3.0 (Takara, Kyoto, Japan), according to the manufacturer's instructions; cDNA was stored at −20°C until needed. The PCR reactions were performed in 25 µl total volume and contained SYBR Premix Ex Taq™ (Takara), as recommended by the manufacturer. The reactions were performed using the 7000 Sequence Detection System (Applied Biosystems, Courtaboeuf, France). Cycling conditions were as follows: one cycle at 95°C for 30 s; 40 cycles at 95°C for 5 s, 55°C for 30 s, and 72°C for 40 s; and one dissociation step of 95°C for 15 s, 60°C for 30 s, and 95°C for 15 s. All samples were analysed in triplicate and normalized against *16S rRNA* expression. Relative expression levels were determined by the (ΔΔCt) method described in Applied Biosystems User Bulletin no. 2 [39].

**Table 2:** Primers used for quantitative RT-PCR in the study.

Primer	Sequence	Location within gene
*16S rRNA*-fw	5′-GCTGCCCTTTGTATTGTC-3′	287–305
*16S rRNA*-rv	5′-AGATGTTGGGTTAAGTCCC-3′	446–465
*hla* -fw	5′-TTGGTGCAAATGTTTC-3′	485–501
*hla*-rv	5′-TCACTTTCCAGCCTACT-3′	569–586
*sea*-fw	5′-ATGGTGCTTATTATGGTTATC-3′	335–356
*sea*-rv	5′-CGTTTCCAAAGGTACTGTATT-3′	477–498
*seb*-fw	5′-TGTTCGGGTATTTGAAGATGG-3′	480–501
*seb*-rv	5′-CGTTTCATAAGGCGAGTTGTT-3′	612–633
*agrA-* fw	5′-TGATAATCCTTATGAGGTGCTT-3′	111–133
*agrA-* rv	5′-CACTGTGACTCGTAACGAAAA-3′	253–274

### Statistical analysis

Experimental data were analyzed using the SPSS 12.0 statistical software. Data were expressed as the mean ± SD. Statistical differences were examined using independent Student t-test. A *p* value less than 0.05 was considered to be statistically significant.
